# Normal weight and waist obesity indicated by increased total body fat associated with all-cause mortality in stage 3–5 chronic kidney disease

**DOI:** 10.3389/fnut.2022.982519

**Published:** 2022-09-16

**Authors:** Feng-Ching Shen, Mei-En Chen, Wei-Tsung Wu, I-Ching Kuo, Sheng-Wen Niu, Jia-Jung Lee, Chi-Chih Hung, Jer-Ming Chang, Shang-Jyh Hwang

**Affiliations:** ^1^Division of Nephrology, Department of Internal Medicine, Kaohsiung Medical University Hospital, Kaohsiung Medical University, Kaohsiung, Taiwan; ^2^Department of Nutrition and Dietetics, Kaohsiung Medical University Hospital, Kaohsiung, Taiwan; ^3^Division of Cardiology, Department of Internal Medicine, Kaohsiung Medical University Hospital, Kaohsiung, Taiwan; ^4^Graduate Institute of Clinical Medicine, College of Medicine, Kaohsiung Medical University, Kaohsiung, Taiwan; ^5^Department of Internal Medicine, Kaohsiung Municipal Ta-Tung Hospital, Kaohsiung Medical University, Kaohsiung, Taiwan; ^6^Regenerative Medicine and Cell Therapy Research Center, Kaohsiung Medical University, Kaohsiung, Taiwan; ^7^Faculty of Renal Care, College of Medicine, Kaohsiung Medical University, Kaohsiung, Taiwan

**Keywords:** obesity paradox, all-cause mortality, chronic kidney diseases, normal weight, normal waist, total body fat, body mass index, waist circumference

## Abstract

Patients with chronic kidney disease (CKD) demonstrate a survival benefit with a high body mass index (BMI); this is the obesity paradox. Central obesity has a higher prognostic value than BMI, even in those with normal weight. Whether total body fat percentage (TBF%) provides more information than BMI and waist circumference (WC) remains unknown. We included 3,262 Asian patients with stage 3–5 CKD and divided these patients by TBF% and waist-to-height ratio (WHtR) quartiles (Q1–Q4). TBF% was associated with BMI, WC, nutritional markers, and C-reactive protein. In all patients, BMI but not TBF% or WHtR demonstrated a survival paradox. In patients with BMI <25 kg/m^2^, but not in those with BMI ≥ 25 kg/m^2^, TBF% Q4 and WHtR Q4 were associated with all-cause mortality, with hazard ratios [HRs; 95% confidence intervals (CIs)] of 2.35 (1.31–4.22) and 1.38 (1.06–1.80), respectively. The HRs of TBF% Q4 for all-cause mortality were 2.90 (1.50–5.58) in patients with a normal WC and 3.81 (1.93–7.50) in patients with normal weight and normal WC (All *P* for interaction < 0.05). In conclusion, TBF% can predict all-cause mortality in patients with advanced CKD and a normal weight, normal WC, or both.

## Introduction

The obesity epidemic is growing worldwide and thus has received major attention (1); a high body mass index (BMI) has been associated with various comorbidities including type II diabetes mellitus, cardiovascular diseases (2), and a reduction in life expectancy (3). BMI is a compelling predictor for all-cause mortality in the general population; studies have demonstrated a J-shaped relationship between BMI and all-cause mortality in 1.46 million Caucasian adults (4) and a U-shaped relationship in 850 thousand East Asian adults (5). By contrast, high BMI is associated with low all-cause mortality in dialysis (6) and advance chronic kidney disease (CKD) (7) populations; this is called the obesity paradox or reverse epidemiology. Interestingly, obesity protects against the negative effect of weight loss on mortality in the CKD population (8). Many hypotheses of the obesity paradox, including the involvement of body structure, body composition, lipid metabolism, and cytokine production, have been proposed (9).

BMI may not be correlated with fat mass; in the CKD population, its use in adiposity assessment can be affected by factors such as fluid overload (10) and sarcopenia (11). Central obesity is associated with metabolic syndrome (MS), particularly among those with a normal body weight (12). Visceral fat accumulation leads to metabolic and cardiovascular disorders through the proinflammatory, atherogenic, and diabetogenic adipokine secretion (13). Compared with BMI, central obesity may afford different diagnostic power in predicting all-cause mortality in the presence of the obesity paradox (14–17). Our previous studies have revealed an association between central obesity and all-cause mortality in the presence of the obesity paradox in patients with stage 3–5 CKD (18, 19).

Central obesity is associated with visceral fat but not directly correlated with total body fat (TBF) percentage (TBF%). Studies have demonstrated that high TBF% is independently associated with high all-cause mortality in the general population (20) and patients with CKD (21, 22). Differentiating fat mass from fat-free mass (23), particularly in patients with sarcopenic obesity or normal weight obesity (NWO) (21), may be difficult. Increased TBF% is positively associated with muscle weakness and related to mortality in CKD patients (24). NWO, defined as normal BMI with high body fat mass, is associated with increased cardiometabolic disease and all-cause mortality risks (25). Whether NWO, indicated by TBF%, is associated with all-cause mortality in the presence of the obesity paradox in advanced CKD populations remains unknown. Moreover, whether this association can be used to differentiate the effects of visceral fat and TBF warrants investigation.

In this study, we hypothesized that TBF% would predict all-cause mortality in a CKD cohort with the obesity paradox and that the effect would be modified by obesity. We tested this hypothesis in patients with stage 3–5 CKD divided into TBF% and waist-to-height ratio (WHtR) quartiles. We also studied the prognostic value of TBF% and WHtR for all-cause mortality in patients with CKD and normal or high BMI and normal or high waist circumference.

## Materials and methods

### Study design and participants

This prospective observational study, the Integrated CKD Care Program in Kaohsiung for Delaying Dialysis, involving two affiliated hospitals of Kaohsiung Medical University in Southern Taiwan, was conducted between November 11, 2002, and May 31, 2009, as described previously (18). Here, we extended the follow-up period to December 31, 2014. The inclusion criterion was stage 1–5 CKD without the receipt of any renal replacement therapy. The exclusion criterion was acute kidney injury, defined as a >50% decrease in glomerular filtration rate (eGFR; calculated using the Modification of Diet in Renal Disease equation) within 3 months. To study the impact of TBF% and BMI on all-cause mortality, the included patients were divided into two BMI groups (cutoff: 25 kg/m^2^) and TBF% quartiles (cutoff: 22.2, 27.4, and 31.9% in men and 27.7, 33.6, and 39.0% in women). Moreover, 65 patients with an extreme BMI (<14.9 or >35.1 kg/m^2^) were excluded, as in our previous study (18). We included 3,262 patients with stage 3–5 CKD and a BMI of 15.0–35.0 kg/m^2^.

All patients provided informed consent to participate. The study protocol was approved by the Institutional Review Board of Kaohsiung Medical University Hospital.

### Collection of demographic, medical, and laboratory data

The baseline variables comprised demographic features (i.e., age, BMI, waist circumference, and sex), medical history [i.e., cardiovascular disease, hypertension, mean blood pressure (BP), diabetes, Charlson comorbidity index, and MS], and laboratory data [i.e., eGFR; urine protein-to-creatinine ratio (Upcr); and hemoglobin, albumin, C-reactive protein (CRP), total cholesterol, and triglyceride levels]. The demographic features formed the baseline record, and the medical history was obtained through a doctor's chart review and interview with patients. BMI was calculated by dividing weight (in kilograms) by the square of height (in square meters). Waist and hip measurements were performed in accordance with the World Health Organization protocol (26). The WHtR is the waist circumference (in centimeters) divided by height (in centimeters). Multiple frequencies bioelectrical impedance analysis measures the change in impedance of alternating low and high-frequency electrical currents, which travel more rapidly through water and lean body mass than through fat body mass. The impedance is used to determine body compositions. The device used in this study was the InBody 230 (Biospace Co Ltd., Korea) with 2 frequencies (20 k and 100 kHz), which was also validated in our previous study (27). The MS components comprised a waist circumference of ≥90 cm in men or ≥80 cm in women; systolic BP of ≥130 mmHg or diastolic BP of ≥85 mmHg or hypertension; high density lipoprotein (HDL) cholesterol of >40 mg/dL in men or >50 mg/dL in women; triglyceride of ≥150 mg/dL; and fasting blood glucose of ≥100 mg/dL or diabetes. We used the Charlson comorbidity index to predict mortality in patients for 17 comorbidities: acute myocardial infarction, congestive heart failure, peripheral vascular disease, cerebral vascular accident, dementia, pulmonary disease, connective tissue disorder, peptic ulcer, liver disease, diabetes, diabetes complications, paraplegia, renal disease, cancer, metastatic cancer, severe liver disease, and human immunodeficiency virus (28). The mean arterial pressure was one-third of averaged systolic BP plus two-thirds of averaged diastolic BP, which were measured 3 months before and after enrollment. Upcr was calculated by dividing urine protein (in milligrams) by urine creatinine (in grams) from a random spot urine sample. Biochemistry measurements were performed during screening and baseline visits and then every 3 months, as per the protocol. The laboratory data from 3 months before baseline to 3 months after baseline were averaged and analyzed.

### Outcomes

All-cause mortality was ascertained by reviewing death certificates, patient charts, or the National Death Index. The models of all-cause mortality included patients who had undergone renal replacement therapy and were censored only at death or the end of follow-up.

### Statistical analysis

The baseline characteristics of all the patients were stratified on the basis of their TBF% and BMI. We used percentages to present categorical variables. Means ± standard deviations denote continuous variables with an approximate normal distribution, and medians and their interquartile ranges indicate continuous variables with a skewed distribution. The differences between groups were examined using the chi-square test for categorical variables and one-way analysis of variance for continuous variables. Cox proportional hazards analysis was used to investigate the relationship of TBF% and BMI with all-cause mortality. Skew-distributed continuous variables were log transformed to attain a normal distribution. Covariates were selected on the basis of their clinical relevance, consistent with our previous study (29). The adjusted covariates were age, sex, eGFR, Upcr log, diabetes, cardiovascular disease, smoking, cancer, severe liver disease, hypertension, hemoglobin, BMI, cholesterol log, glycosylated hemoglobin, albumin, CRP ln, and phosphorus.

All statistical analyses were performed on SPSS for Windows (version 20.0; IBM, Chicago, IL, USA).

## Results

### Baseline characteristics of patients with stage 3–5 CKD stratified by BMI and TBF%

The 3,262 included patients were divided into two BMI groups: <25 and ≥25 kg/m^2^ (Table 1). Of these patients, 42% were women, 44.4% had cardiovascular disease, 66.1% had hypertension, 50.3% had diabetes, and 49.5% had MS. The mean age was 63.5 ± 13.4 years, mean eGFR was 22.4 ± 11.2 mL/min/1.73 m^2^, and mean Upcr was 1,110 (interquartile range: 405–2,541) mg/g. Compared with patients with BMI <25 kg/m^2^, more patients with BMI ≥ 25 kg/m^2^ had diabetes, MS, and a higher BMI, waist circumference, mean BP, eGFR, hemoglobin level, CRP level, and triglyceride level. At follow-up, 43% of patients developed end-stage renal disease (ESRD), and 27.6% patients died. Compared with patients with BMI <25 kg/m^2^, more patients with BMI ≥ 25 kg/m^2^ progressed to ESRD and all-cause mortality.

**Table 1:** Baseline characteristics of patients with stage 3–5 chronic kidney disease stratified by body mass index.

	**Body mass index**		***P*-value**
	**(Kg/m** ^**2**^ **)**		**(ANOVA)**
	** <25**	**≥25**	
No. of patients (*n* = 3,262)	1,875	1,387	
**Demographics/medical history**		
Age (years)	63.8 (±13.7)	63.2 (±13.1)	0.177
BMI (kg/m^2^)	22.1 (±2.1)	27.9 (±2.3)	<0.001
Waist (cm)	82.5 (±12.0)	95.0 (±11.6)	<0.001
Sex (female)	830 (44.3%)	539 (38.8%)	0.002
Cardiovascular disease	461 (24.6%)	386 (27.8%)	0.038
Hypertension	1,195 (63.7%)	962 (69.3%)	0.001
Mean BP (mmHg)	98.8 (±13.7)	101.3 (±13.6)	<0.001
Diabetes mellitus	873 (46.6%)	768 (55.3%)	<0.001
Charlson score	3.5 (±2.1)	3.6 (±2.0)	0.201
Metabolic syndrome	1,073 (57.2%)	1,196 (86.1%)	<0.001
**Laboratory data**		
eGFR (ml/min/1.73 m^2^)	23.3 (±15.0)	26.6 (±15.1)	<0.001
Upcr (mg/g)	1,182 (436–2,669)	1,006 (374–2,413)	0.008
Hemoglobin (g/dl)	10.5 (±2.2)	11.5 (±2.4)	<0.001
Albumin (g/dl)	3.8 (±0.5)	3.9 (±0.5)	0.009
C-reactive protein (mg/l)	1.0 (0.4–4.5)	1.4 (0.5–6.6)	<0.001
Total cholesterol (mg/dl)	190 (160–221)	192 (165–222)	0.095
Triglyceride (mg/dl)	115 (83–165)	143.0 (104–206)	<0.001
**Outcomes**		
ESRD	851 (45.4%)	551 (39.7%)	0.002
All-cause mortality	554 (29.5%)	346 (24.9%)	0.004

Data are presented as mean (standard error), median (interquartile range), or number (%).

BMI, body mass index; eGFR, estimated glomerular filtration rate; Upcr, urine protein and creatinine ratio; ESRD, end-stage renal disease.

Of all 3,262 patients with stage 3–5 CKD, 1,237 received simultaneous body composition analysis with bioelectrical impedance spectroscopy. The participants were divided into TBF% quartiles on the basis of their BMI (<25 and ≥25 kg/m^2^; Table 2). In the <25 kg/m^2^ BMI group, age, BMI, WHtR, cardiovascular disease, MS, eGFR, hemoglobin level, albumin level, CRP level, triglyceride level, and all-cause mortality increased with TBF%, but progression to ESRD and Upcr decreased. In the ≥25 kg/m^2^ BMI group, age, BMI, WHtR, DM, eGFR, hemoglobin level, and albumin level increased with TBF%, but progression to ESRD and Upcr decreased; however, all-cause mortality did not reach statistical significance.

**Table 2:** Baseline characteristics of patients with stage 3–5 chronic kidney disease stratified by total body fat and body mass index.

	**Total body fat (%)**				***P*-value (ANOVA)**
	**Q1**	**Q2**	**Q3**	**Q4**	
Male	<22.2%	22.2–27.4%	27.4–31.9%	>31.9%	
Female	<27.7%	27.7–33.6%	33.6–39.0%	>39.0%	
**BMI** **<** **25 (Kg/m**^**2**^**)**					
No. of patients (*n* = 701)	*n* = 268 (38.2%)	*n* = 221 (31.5%)	*n* = 151 (21.5%)	*n* = 61 (8.7%)	
**Demographics/medical history**					
Age (years)	59.6 (±14.0)	63.2 (±11.4)	65.6 (±11.9)	68.9 (±11.9)	<0.001
BMI (kg/m^2^)	20.9 (±2.2)	22.3 (±1.9)	23.2 (±1.4)	23.3 (±1.9)	<0.001
WHtR (%)	47.5 (±4.9)	50.5 (±4.3)	52.2 (±3.6)	53.1 (±5.2)	<0.001
Sex (Female)	111 (41.4%)	99 (44.8%)	65 (43.0%)	23 (37.7%)	0.754
Cardiovascular disease	42 (15.7%)	32 (14.5%)	35 (23.2%)	17 (27.9%)	0.022
Hypertension	189 (70.5%)	149 (67.4%)	98 (64.9%)	46 (75.4%)	0.411
Mean BP (mmHg)	100 (±15.4)	98.8 (±16.6)	98.7 (±14.6)	97.7 (±14.8)	0.596
Diabetes mellitus	94 (35.1%)	71 (32.1%)	53 (35.1%)	23 (37.7%)	0.828
Charlson score	3.2 (±2.0)	3.0 (±2.1)	3.5 (±2.7)	3.7 (±2.3)	0.087
Metabolic syndrome	73 (27.2%)	68 (30.8%)	66 (43.7%)	31 (50.8%)	<0.001
**Laboratory data**					
eGFR (ml/min/1.73 m^2^)	22.6 (±15.4)	25.7 (±15.8)	26.7 (±14.9)	29.1 (±14.7)	0.005
Upcr (mg/g)	1,098 (452–2,426)	851 (302–1,862)	604 (258–1,479)	473 (196–1,332)	<0.001
Hemoglobin (g/dl)	10.4 (±2.1)	11.0 (±2.0)	11.1 (±2.0)	11.7 (±1.9)	<0.001
Albumin (g/dl)	3.9 (±0.6)	4.1 (±0.4)	4.1 (±0.4)	4.0 (±0.4)	<0.001
C-reactive protein (mg/l)	0.8 (0.3–2.8)	1.0 (0.5–3.1)	1.2 (0.6–4.3)	1.8 (0.8–5.6)	0.001
Total cholesterol (mg/dl)	182 (154–207)	187 (164–213)	192 (163–219)	191 (167–209)	0.049
Triglyceride (mg/dl)	92 (66–133)	110 (78–156)	116 (88–160)	126 (94–166)	<0.001
**Outcomes**					
ESRD	142 (45.5%)	94 (30.1%)	62 (19.9%)	14 (4.5%)	<0.001
All-cause mortality	48 (17.9%)	34 (15.4%)	30 (19.9%)	22 (36.1%)	0.004
**BMI** **≥25 (Kg/m**^**2**^**)**					
No. of patients (*n* = 536)	*n* = 40 (7.5%)	*n* = 89 (16.6%)	*n* = 159 (29.7%)	*n* = 248 (46.3%)	
**Demographics/medical history**					
Age (years)	55.5 (±13.2)	58.9 (±12.9)	61.3 (±12.3)	63.0 (±12.9)	0.001
BMI (kg/m^2^)	27.1 (±2.1)	26.8 (±1.7)	27.5 (±1.8)	28.7 (±2.4)	<0.001
WHtR (%)	55.1 (±3.8)	55.7 (±4.7)	57.0 (±4.3)	59.8 (±4.6)	<0.001
Sex (female)	13 (32.5%)	26 (29.2%)	60 (37.7%)	102 (41.1%)	0.219
Cardiovascular disease	11 (27.5%)	18 (20.2%)	34 (21.4%)	62 (25.0%)	0.661
Hypertension	35 (87.5%)	71 (79.8%)	127 (79.9%)	202 (81.5%)	0.718
Mean BP (mmHg)	103.7 (±17.9)	106.0 (±18.1)	100.8 (±15.3)	101.1 (±17.0)	0.067
Diabetes mellitus	26 (65.0%)	37 (41.6%)	73 (45.9%)	134 (54.0%)	0.032
Charlson score	3.6 (±2.2)	3.2 (±2.3)	3.3 (±2.1)	3.6 (±2.3)	0.423
Metabolic syndrome	25 (62.5%)	60 (67.4%)	112 (70.4%)	191 (77.0%)	0.1108
**Laboratory data**					
eGFR (ml/min/1.73 m^2^)	17.7 (±12.1)	28.1 (±16.8)	29.6 (±15.9)	30.4 (±16.3)	<0.001
Upcr (mg/g)	2,636 (1,084–6,273)	1,318 (481–2,655)	654 (257–1,778)	879 (265–1,855)	<0.001
Hemoglobin (g/dl)	10.4 (±2.0)	11.6 (±2.4)	12.0 (±2.4)	11.9 (±2.3)	0.001
Albumin (g/dl)	3.6 (±0.6)	4.0 (±0.5)	4.1 (±0.4)	4.1 (±0.4)	<0.001
C-reactive protein (mg/l)	2.3 (0.6–6.7)	1.3 (0.4–3.9)	1.3 (0.5–3.4)	1.6 (0.6–5.2)	0.144
Total cholesterol (mg/dl)	186 (150–221)	186 (162–216)	182 (157–212)	193 (166–220)	0.288
Triglyceride (mg/dl)	129 (92–168)	147 (96–204)	131 (103–201)	143 (108–213)	0.339
**Outcomes**					
ESRD	32 (80.0%)	39 (43.8%)	59 (37.1%)	84 (33.8%)	<0.001
All-cause mortality	9 (22.5%)	12 (13.5%)	17 (10.7%)	40 (16.1%)	0.211

Data are presented as means (standard errors), medians (interquartile ranges), or numbers (%).

BMI, body mass index; WHtR, waist-to-height ratio; eGFR, estimated glomerular filtration rate; Upcr, urine protein and creatinine ratio; ESRD, end-stage renal disease.

### Linear regression for total body fat percentage by BMI

The univariate linear regression for TBF% (Supplementary Table 1) and multivariate linear regression for TBF% (Supplementary Table 2) revealed a significant relationship between TBF% and age, gender, Upcr log, BMI, waist, hemoglobin, triglyceride log, albumin, and CRP ln. We further analyzed the association between TBF% and variants according to BMI subgroups (BMI <25 kg/m^2^ and BMI ≥ 25 kg/m^2^) (Table 3). Our results showed that TBF% is significantly associated with age, gender, BMI, albumin, hemoglobin, triglyceride log, and CRP ln in normal weight (BMI <25 kg/m^2^) patients; TBF% is significantly associated with age, gender, BMI, albumin, and Upcr log in preobese-obese (BMI ≥ 25 kg/m^2^) patients.

**Table 3:** Linear regression for total body fat percentage by BMI <25 kg/m^2^ and BMI ≥ 25 kg/m^2^ (per 10% increase).

	**Total body fat (%)**			
	**Normal weight**		**Preobese-obese**	
	**(BMI**<**25 kg/m**^**2**^**)**		**(BMI** ≥**25 kg/m**^**2**^**)**	
	**Beta coefficient (95% CI)**	***P*-value**	**Beta coefficient (95% CI)**	***P*-value**
Age (years)	0.112 (0.076–0.149)	<0.001	0.108 (0.066–0.149)	<0.001
Gender (female vs. male)	8.726 (7.680–9.772)	<0.001	8.078 (6.968–9.188)	<0.001
BMI (kg/m^2^)	1.393 (1.163–1.623)	<0.001	1.235 (1.001–1.468)	<0.001
Albumin (g/dl)	3.558 (2.572–4.544)	<0.001	3.560 (2.225–4.895)	<0.001
Upcr log			−1.301 (−2.323 to −0.279)	0.013
Hemoglobin (g/dl)	0.443 (0.188–0.698)	0.001		
Triglyceride log	1.479 (0.241–2.716)	<0.001		
CRP ln	1.185 (0.563–1.807)	<0.001		

BMI, body mass index; CI, confidence interval; BMI, body mass index; Upcr, urine protein-to-creatinine ratio; CRP, C-reactive protein.

### Association between TBF%, WHtR, and all-cause mortality stratified by BMI

**Figure 1:**
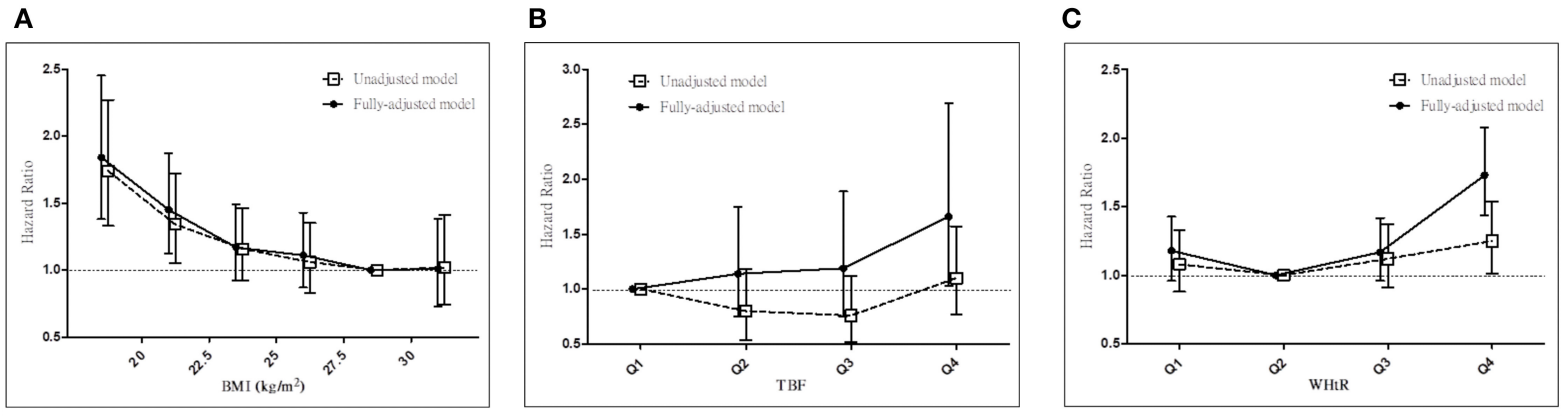
Associations between anthropometric markers and all-cause mortality in all patients with stage 3–5 CKD. Hazard ratios for all-cause mortality based on **(A)** BMI, **(B)** total body fat percentage, and **(C)** WHtR before and after adjustments. CKD, chronic kidney disease; BMI, body mass index; WHtR, waist-to-height ratio.

In the entire cohort, we noted the BMI paradox but not the TBF% or WHtR paradoxes for all-cause mortality in our fully adjusted Cox regression model. High BMI was associated with low all-cause mortality (Figure 1A), whereas high TBF% (Figure 1B) and WHtR (Figure 1C) were associated with high all-cause mortality in our patients with stage 3–5 CKD. Therefore, TBF% and WHtR might be better indicators for obesity.

**Figure 2:**
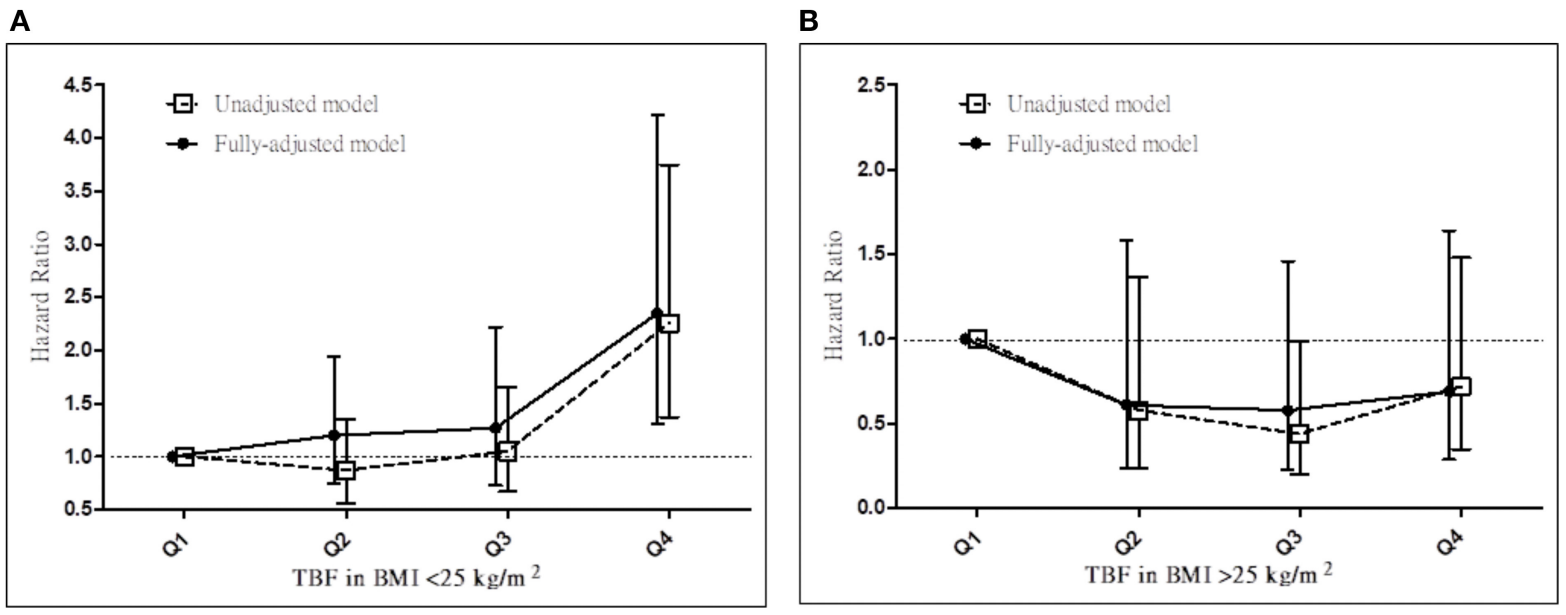
Hazard ratios for all-cause mortality based on total body fat percentage in patients with a BMI of **(A)** <25 and **(B)** ≥25 kg/m^2^ before and after adjustments. TBF%, total body fat percentage; BMI, body mass index.

We next determined whether the prognostic effect of TBF% on all-cause mortality differs between the <25 and ≥25 kg/m^2^ BMI groups (Table 4A; Figures 2A,B). In the <25 kg/m^2^ BMI group, HRs (95% CI) for all-cause mortality increased significantly in the TBF% Q4 group [2.35 (1.31–4.22)] and marginally in the TBF% Q2 [1.20 (0.74–1.95)] and Q3 [1.27 (0.73–2.22)] groups compared with the reference group (TBF% Q1). In the ≥25 kg/m^2^ BMI group, HRs (95% CI) for all-cause mortality non-significantly decreased in the TBF% Q2 [0.61 (0.24–1.58)], Q3 [0.58 (0.23–1.46)], and Q4 [0.69 (0.29–1.64)] groups compared with the reference group (TBF% Q1; *P* for interaction = 0.009).

**Table 4A:** Hazard ratios for all-cause mortality based on total body fat percentage and body mass index.

		**Total body fat (%)**			
**HR for all-cause mortality**		**Q1**	**Q2**	**Q3**	**Q4**
	Male	<22.2%	22.2–27.4%	27.4–31.9%	>31.9%
	Female	<27.7%	27.7–33.6%	33.6–39.0%	>39.0%
BMI <25 (Kg/m^2^)	Unadjusted	1 (reference)	0.87 (0.56–1.35)	1.05 (0.67–1.66)	2.26 (1.37–3.75)*
	Fully-adjusted	1 (reference)	1.20 (0.74–1.95)	1.27 (0.73–2.22)	2.35 (1.31–4.22)*
BMI ≥ 25 (Kg/m^2^)	Unadjusted	1 (reference)	0.58 (0.24–1.37)	0.44 (0.20–0.99)*	0.72 (0.35–1.48)
	Fully-adjusted	1 (reference)	0.61 (0.24–1.58)	0.58 (0.23–1.46)	0.69 (0.29–1.64)

Values are expressed as hazard ratios and 95% confidence intervals.

Fully adjusted model, adjusted for age, sex, eGFR, Upcr log, diabetes, cardiovascular disease, smoking, cancer, severe liver disease, hypertension, hemoglobin, body mass index, cholesterol log, glycosylated hemoglobin, albumin, CRP ln, and phosphorus.

^*^P < 0.05 compared with reference TBF% group.

HR, hazard ratio; BMI, body mass index; eGFR, estimated glomerular filtration rate; Upcr, urine protein and creatinine ratio; CRP, C-reactive protein; TBF%, total body fat percentage.

We also determined whether the prognostic effect of WHtR on all-cause mortality differs between the <25 and ≥25 kg/m^2^ BMI groups (Table 4B). In the <25 kg/m^2^ BMI group, HRs (95% CIs) for all-cause mortality increased significantly in the WHtR Q3 [1.31 (1.02–1.68)] and Q4 [1.38 (1.06–1.80)] groups compared with the reference group (WHtR Q2). In the ≥25 kg/m^2^ BMI group, HRs (95% CIs) for all-cause mortality increased significantly in the WHtR Q1 group [1.68 (1.10–2.57)] and marginally in the WHtR Q4 [1.24 (0.95–1.63)] and Q2 [1.28 (0.88–1.86)] groups compared with the reference group (WHtR Q3; *P* for interaction = 0.206).

**Table 4B:** Hazard ratios for all-cause mortality based on waist-to-height ratio and body mass index.

		**Waist-to-height ratio**			
**HR for all-cause mortality**		**Q1**	**Q2**	**Q3**	**Q4**
	Male	<50.0%	50.0–54.3%	54.3–58.9%	>58.9%
	Female	<49.0%	49.0–54.9%	54.9–61.0%	>61.0%
BMI <25 (Kg/m^2^)	Unadjusted	1.09 (0.87–1.36)	1 (reference)	1.53 (1.20–1.95)*	2.74 (2.13–3.51)*
	Fully-adjusted	1.05 (0.83–1.32)	1 (reference)	1.31 (1.02–1.68)*	1.38 (1.06–1.80)*
BMI ≥ 25 (Kg/m^2^)	Unadjusted	1.50 (1.00–2.26)	0.99 (0.69–1.42)	1 (reference)	1.70 (1.32–2.19)*
	Fully-adjusted	1.68 (1.10–2.57)*	1.28 (0.88–1.86)	1 (reference)	1.24 (0.95–1.63)

Values are expressed as hazard ratios and 95% confidence intervals.

Fully adjusted model, adjusted for age, sex, eGFR, Upcr log, diabetes, cardiovascular disease, smoking, cancer, severe liver disease, hypertension, hemoglobin, body mass index, cholesterol log, glycosylated hemoglobin, albumin, CRP ln, and phosphorus.

^*^P < 0.05 compared with reference WHtR group.

HR, hazard ratio; BMI, body mass index; eGFR, estimated glomerular filtration rate; Upcr, urine protein and creatinine ratio; CRP, C-reactive protein; WHtR, waist-to-height ratio.

### Association between TBF% and all-cause mortality stratified by WHtR

Both TBF% and WHtR were noted to be indicators for NWO according to our results in the <25 kg/m^2^ BMI group. To explore the prognostic value of TBF% in patients with CKD, we determined whether HRs of TBF% for all-cause mortality differ between the high– and low–waist circumference groups (Table 5). In the normal–waist circumference group (waist circumference < MS criteria), the HRs (95% CI) for all-cause mortality increased significantly in the TBF% Q4 group [2.90 (1.50–5.58)] but marginally in the TBF% Q2 group [1.24 (0.73–2.10)] compared with the reference group (TBF% Q1). In the high–waist circumference group (waist circumference ≥ MS criteria), the HRs (95% CIs) for all-cause mortality decreased marginally in the TBF% Q3 [1.60 (0.70–3.67)] and Q4 [1.44 (0.63–3.28)] groups compared with the reference group (TBF% Q1; *P* for interaction = 0.038).

**Table 5:** Hazard ratios for all-cause mortality based on total body fat percentage and waist circumference.

		**Total body fat (%)**			
**HR for all-cause mortality**		**Q1**	**Q2**	**Q3**	**Q4**
	Male	<22.2%	22.2–27.4%	27.4–31.9%	>31.9%
	Female	<27.7%	27.7–33.6%	33.6–39.0%	>39.0%
Normal waist^#^	Unadjusted	1 (reference)	0.78 (0.49–1.25)	0.68 (0.39–1.17)	1.90 (1.12–3.21)*
	Fully-adjusted	1 (reference)	1.24 (0.73–2.10)	1.03 (0.55–1.96)	2.90 (1.50–5.58)*
Increased waist^+^	Unadjusted	1 (reference)	0.70 (0.34–1.45)	0.66 (0.34–1.29)	0.69 (0.37–1.32)
	Fully-adjusted	1 (reference)	1.15 (0.50–2.68)	1.60 (0.70–3.67)	1.44 (0.63–3.28)

^#^Normal waist circumference: waist circumference < metabolic syndrome criteria.

^+^Increased waist circumference: waist circumference ≥ metabolic syndrome criteria; Metabolic syndrome criteria: waist circumference ≥ 90 cm in men or ≥ 80 cm in women.

Values are expressed as hazard ratios and 95% confidence intervals.

Fully adjusted model, adjusted for age, sex, eGFR, Upcr log, diabetes, cardiovascular disease, smoking, cancer, severe liver disease, hypertension, hemoglobin, body mass index, cholesterol log, glycosylated hemoglobin, albumin, CRP ln, and phosphorus.

*P < 0.05 compared with reference TBF% group.

HR, hazard ratio; BMI, body mass index; eGFR, estimated glomerular filtration rate; Upcr, urine protein and creatinine ratio; CRP, C-reactive protein; WHtR, waist-to-height ratio; TBF%, total body fat percentage.

### Association between TBF% and all-cause mortality stratified by BMI and WHtR

Because TBF% is an indicator for normal waist obesity in the normal–waist circumference group and because BMI and waist circumference are closely related, we explored the prognostic value of TBF% in patients with or without normal BMI and normal waist circumference (Table 6). In the <25 kg/m^2^ BMI and normal–waist circumference group, the HRs (95% CIs) for all-cause mortality increased significantly in the TBF% Q4 group [3.81 (1.93–7.50)] and marginally in the TBF% Q2 [1.22 (0.70–2.13)] and Q3 [1.21 (0.62–2.36)] groups compared with the reference group (TBF% Q1). In the ≥25 kg/m^2^ BMI or increased–waist circumference group, the HR for all-cause mortality was not associated with any of the TBF% groups compared with the reference group (TBF% Q1; *P* for interaction = 0.012).

**Table 6:** Hazard ratios for all-cause mortality based on total body fat percentage, body mass index and waist circumference.

		**Total body fat (%)**			
**HR for all-cause mortality**		**Q1**	**Q2**	**Q3**	**Q4**
	Male	<22.2%	22.2–27.4%	27.4–31.9%	>31.9%
	Female	<27.7%	27.7–33.6%	33.6–39.0%	>39.0%
BMI <25 and normal waist^#^	Unadjusted	1 (reference)	0.81 (0.49–1.34)	0.89 (0.51–1.57)	3.03 (1.70–5.41)*
	Fully-adjusted	1 (reference)	1.22 (0.70–2.13)	1.21 (0.62–2.36)	3.81 (1.93–7.50)*
BMI ≥25 or increased waist^+^	Unadjusted	1 (reference)	0.65 (0.34–1.23)	0.54 (0.30–1.00)*	0.66 (0.38–1.17)
	Fully-adjusted	1 (reference)	0.93 (0.44–1.94)	1.01 (0.49–2.09)	1.03 (0.50–2.12)

^#^Normal waist circumference: waist circumference < metabolic syndrome criteria.

^+^Increased waist circumference: waist circumference ≥ metabolic syndrome criteria; Metabolic syndrome criteria: waist circumference ≥ 90 cm in men or ≥80 cm in women.

Values are expressed as hazard ratios and 95% confidence intervals.

Fully adjusted model, adjusted for age, sex, eGFR, Upcr log, diabetes, cardiovascular disease, smoking, cancer, severe liver disease, hypertension, hemoglobin, body mass index, cholesterol log, glycosylated hemoglobin, albumin, CRP ln, and phosphorus.

*P < 0.05 compared with reference TBF% group.

HR, hazard ratio; BMI, body mass index; eGFR, estimated glomerular filtration rate; Upcr, urine protein and creatinine ratio; CRP, C-reactive protein; WHtR, waist-to-height ratio; TBF%, total body fat percentage.

### HR of NWO for all-cause mortality based on TBF% and WHtR

We determined the cutoff for NWO and non-NWO on the basis of TBF% or WHtR (Supplementary Table 3). When defined by TBF% Q3 and WHtR Q3, the HRs (95% CIs) of NWO for all-cause mortality compared with preobese obesity were 1.73 (1.14–2.64) and 1.34 (1.13–1.59), respectively.

### Association between TBF% and MS stratified by BMI

We determined the association between MS prevalence and TBF% by using a fully adjusted logistic regression model (Supplementary Table 4). Compared with the reference group (TBF% Q1), MS prevalence increased with TBF% significantly in the <25 kg/m^2^ BMI group {odds ratios [ORs; 95% confidence intervals (CIs)] of TBF% Q3 and TBF% Q4: 2.01 (1.25–3.22) and 2.33 (1.24–4.37), respectively} and marginally in the ≥25 kg/m^2^ BMI group [ORs (95% CIs) of TBF% Q3 and TBF% Q4: 1.57 (0.69–3.59) and 2.01 (0.90–4.52), respectively; *P* for interaction = 0.359].

## Discussion

In our advanced CKD cohort, we noted the obesity paradox (Figure 1A) but not the TBF% (Figure 1B) or WHtR (Figure 1C) paradoxes. Moreover, TBF% predicted all-cause mortality in our patients with normal BMI (Table 4A; Figure 2A) but not in those with high BMI (Table 4A; Figure 2B), whereas WHtR predicted all-cause mortality in the obesity group (Table 4B). Notably, TBF% predicted all-cause mortality not only in patients with normal weight but also in those with normal waist circumference (Table 5). We observed that TBF% predicted all-cause mortality in patients with normal weight and waist circumference (Table 6). Therefore, TBF% is a potential indicator for obesity in patients with advanced CKD, especially in those with normal weight, normal waist circumference, or both.

BMI is a strong predictor for all-cause mortality in the general population. Both high and low BMI are associated with increased all-cause mortality; the lowest all-cause mortality has been noted in Caucasian adults with a BMI of 20.0–24.9 kg/m^2^ (4) and East Asian adults with a BMI of 22.6–27.5 kg/m^2^ (5). Evidence has demonstrated that all-cause mortality is low in obese and overweight groups of specific populations; this is the obesity paradox (30). The relationship was initially reported by Gruberg et al. in patients with coronary artery disease after percutaneous coronary intervention (31); many studies thereafter have revealed a protective effect due to high BMI in patients under maintenance dialysis (32), with chronic heart failure (33), after acute myocardial infarction (34), with a chronic obstructive lung disease (35), and who are nursing home residents (36). The current study revealed the obesity paradox for all-cause mortality; high BMI was associated with low all-cause mortality in our advanced CKD population—consistent with the results of other studies (16, 17), including those of a metanalysis including 484,906 patients with stage 3–5 CKD (37).

Evidence has suggested that BMI is inadequate for body composition measurement and that additional anthropometric markers should be used for determining body fat mass (38), particularly in CKD populations (23). The association between excess visceral fat accumulation and MS has been observed, and several hypothesis have been proposed (12). Increased visceral fat levels can enhance the delivery of free fatty acids to the liver and contribute to insulin resistance in the liver and subsequently to muscular tissues (39). Studies have suggested that visceral fat alters the secretion of various adipokines, including adiponectin, interleukin 6, tumor necrosis factor alpha, CRP, plasminogen activator inhibitor 1, and resistin, which have a potent effect on adipose tissue metabolism (40, 41). MS, also known as syndrome X or insulin resistance syndrome, is associated with increased cardiovascular disease risks (42). Studies have demonstrated that central obesity, indicated by waist circumference (14, 17), waist-to-hip ratio (14–16, 43), WHtR (44, 45), and conicity index (19, 46), provides higher diagnostic power than does BMI alone for the evaluation of all-cause mortality in CKD populations. Our study revealed that central obesity is associated with all-cause mortality in both high- and low-WHtR groups (Figure 1C).

NWO is associated with a high prevalence of cardiometabolic dysregulation, MS, and cardiovascular risks (47). Individuals with metabolically NWO, first described in the late 1990s, have been noted to be insulin resistant and predisposed to type 2 diabetes mellitus, hypertriglyceridemia, and premature coronary heart disease (48). Low lean mass and high fat mass are common among patients with CKD (49) and associated with increased all-cause mortality (50, 51). Body fat distribution and high levels of body fat are both strongly related to morbidity and all-cause mortality in CKD populations (52). Misclassification of obese populations as non-obese based on TBF% and BMI is common in both non–dialysis-dependent (21, 23) and dialysis-dependent (53) patients with CKD.

Whether the metabolic derangement in individuals with NWO arises from increased TBF% or a centralized body fat distribution in CKD populations remains unknown. Indexes of central fatness including waist circumference, waist-to-hip ratio, and WHtR are positively and significantly associated with increased all-cause mortality risk (54). In adults, WHtR demonstrates superiority over waist circumference and BMI for cardiometabolic risk factor detection (55). The current results reveal that high WHtR is significantly associated with all-cause mortality in patients with normal body weight. By contrast, high TBF% is associated with elevated all-cause mortality in patients with CKD (21); this relationship has been noted to be J shaped in the general population (22). Muscle weakness, measured by hand grip strength and pinch strength, are associated with increased mortality in CKD population and weaker hand grip strength is noticed in CKD patients with increased fat mass (24). Lin et al. reported a significantly higher all-cause mortality on the basis of TBF% and lower all-cause mortality on the basis of BMI in an obese CKD population (21). Studies have also demonstrated that ~25% of non–dialysis-dependent patients with CKD (23) and 55% of dialysis-dependent patients with CKD (53) receive misdiagnoses of non-obesity when BMI is used as the anthropometric marker rather than TBF%. Furthermore, weight loss is associated with mortality in the CKD population (56) and confounded the association between obesity and mortality. Sarcopenic patients were protected by adiposity due to its ability to maintain weight; therefore, CKD patients with higher fat mass have greater survival benefit among those with lower muscle mass (8). Our results demonstrate that high TBF% is strongly associated with MS and all-cause mortality in patients with normal body weight—corroborating previous results (47, 57).

To evaluate the prognostic value of TBF% for all-cause mortality, we analyzed the results on the basis of the different waist circumference and weight groups. NWO is a well-recognized predictor for all-cause mortality; however, the effects of normal waist obesity on all-cause mortality remain unknown. Individuals with normal waist circumference have a low MS risk (58) as well as low all-cause mortality (17, 59), independent of BMI, compared with those with increased waist circumference. The association of decreased all-cause mortality with normal waist circumference has also been reported in CKD populations (15). Several studies have focused on the effects of NWO on all-cause mortality, but only a few have discussed the effects of excess body fat in individuals with normal waist circumference. BMI cannot differentiate TBF from lean mass and central fat from peripheral fat (60). Although waist circumference can predict abdominal obesity (61), it has a limited effect on TBF prediction. Our results reveal a significantly increased all-cause mortality in high-TBF% patients with normal weight, normal waist circumference, or both. Therefore, these results demonstrate the prognostic value of TBF%.

No consensus regarding the method of defining obesity by TBF% is available. The American Society of Endocrinologists defines obesity as TBF% of >35% in women and >25% in men (62). De Lorenzo et al. (63) described the relationship between body fat distribution and cardiovascular disease risk in individuals with NWO, defined as normal BMI (<25 kg/m^2^) with increased TBF% (>30%). Healthy TBF% may be defined on the basis of BMI, sex, and age (64). A study reported that the TBF% cutoff for NWO is 20.2–28.2% in men and 29.9–39.6% in women on the basis of age (65); another study defined NWO to be the highest tertile of TBF% Q4 (>23.1% in men and >33.3% in women) (47). Our results provide clinical reference for NWO based on TBF% in advanced CKD populations. Here, the highest quartile of TBF% (>30.5% in men and >37.1% in women) was labeled as NWO. The cutoff was higher than that reported previously, probably because of the obesity paradox and ethnic differences.

The main strength of this study is the large sample size (*N* = 3,262) as well as the enrollment of patients with stage 3–5 CKD and BMI of 15.0–35.0 kg/m^2^. To the best of our knowledge, this is the first study focusing on the all-cause mortality of advance-stage CKD populations with normal weights and normal waist circumferences stratified by TBF%. This study also has several limitations. First, the baseline anthropometric measurements were used for analysis, and we did not estimate the time-dependent changes. Second, the Integrated CKD Care Program Kaohsiung for Delaying Dialysis is a sample of an East Asian population; therefore, we could not address the importance of ethnicity in body composition or outcome. Third, we did not include dietary and medication factors in our study, and the importance of their effects on obesity and CKD incidence should be considered. Fourth, our patients only had advanced CKD; thus, the current results may not be applicable to all CKD populations. Additional studies clarifying the nature of the obesity paradox and focusing on different anthropometric markers in the prediction of all-cause mortality, particularly in advanced CKD patients with normal weight and normal waist circumference, are warranted.

## Conclusion

This study revealed that patients with advanced CKD demonstrate the BMI paradox but not the WHtR or TBF% paradoxes. High TBF% is associated with increased all-cause mortality in those with normal weight, normal waist circumference, or both. TBF% is a good indicator for NWO, normal waist obesity, or both in the advanced CKD population.

## Data availability statement

The original contributions presented in the study are included in the article/Supplementary material, further inquiries can be directed to the corresponding author/s.

## Ethics statement

The studies involving human participants were reviewed and approved by Institutional Review Board of Kaohsiung Medical University Hospital. The patients/participants provided their written informed consent to participate in this study.

## Author contributions

F-CS and C-CH: conceptualization, formal analysis, methodology, and writing—original draft. J-MC and S-JH: supervision. M-EC, W-TW, I-CK, S-WN, and J-JL: writing—review and editing. All authors have read and agreed to the published version of the manuscript.

## Conflict of interest

The authors declare that the research was conducted in the absence of any commercial or financial relationships that could be construed as a potential conflict of interest.

## Publisher's note

All claims expressed in this article are solely those of the authors and do not necessarily represent those of their affiliated organizations, or those of the publisher, the editors and the reviewers. Any product that may be evaluated in this article, or claim that may be made by its manufacturer, is not guaranteed or endorsed by the publisher.
