# Supplementary material for: Normal weight and waist obesity indicated by increased total body fat associated with all-cause mortality in stage 3–5 chronic kidney disease
Source: Front Nutr. 2022 Sep 16;9:982519. doi: 10.3389/fnut.2022.982519 (PMC9523665; doi:10.3389/fnut.2022.982519)
Supplement: Supplementary file 1 [file Data_Sheet_1.pdf]

Supplementary Material

Supplementary Table 1 | Univariate linear regression for total body fat percentage (per 10% increase)

	Beta coefficient	95% CI Beta coefficient	*P* value
Age (years)	0.073	0.035 to 0.112	<0.001
Gender (female vs male)	6.449	5.498 to 7.399	<0.001
eGFR (ml/min/1.73 m^2)	0.034	0.003 to 0.065	0.033
Upcr log	-1.243	-2.094 to -0.393	0.004
Diabetes mellitus	1.179	0.169 to 2.190	0.022
Hypertension	1.273	0.136 to 2.410	0.028
Cardiovascular disease	1.479	0.241 to 2.716	0.019
Charlson score	0.194	-0.030 to 0.418	0.089
Waist (cm)	0.295	0.240 to 0.340	<0.001
Mean blood pressure (mmHg)	-0.014	-0.045 to 0.017	0.376
Glycosylated hemoglobin (%)	0.679	0.303 to 1.055	<0.001
Hemoglobin (g/dl)	0.347	0.124 to 0.569	0.002
Triglyceride log	9.899	7.824 to 11.974	<0.001
Albumin (g/dl)	3.803	2.776 to 4.829	<0.001
CRP ln	1.030	0.353 to 1.706	0.003
Phosphorus (mg/dl)	-0.677	1.106 to -0.249	0.002

Abbreviations: CI, confidence interval; eGFR, estimated glomerular filtration rate; Upcr, urine protein and creatinine ratio; CRP, C-reactive protein.

Supplementary Table 2 | Multivariate linear regression for total body fat percentage (per 10% increase)

	Beta coefficient	95% CI Beta coefficient	*P* value
Age (years)	0.104	0.0075 to 0.134	<0.001
Gender (female vs male)	8.856	8.006 to 9.707	<0.001
Upcr log	-0.862	-1.619 to -0.105	0.026
BMI (kg/m^2)	1.122	0.955 to 1.290	<0.001
Waist (cm)	0.074	0.011 to 0.137	0.021
Hemoglobin (g/dl)	0.256	0.063 to 0.450	0.010
Triglyceride log	3.265	1.659 to 4.872	<0.001
Albumin (g/dl)	3.407	2.553 to 4.261	<0.001
CRP ln	0.605	0.126 to 1.085	0.013

Abbreviations: CI, confidence interval; eGFR, estimated glomerular filtration rate; CRP, C-reactive protein.

Supplementary Table 3 | HR of NOW for all-cause mortality based on TBF% and WHtR

**HR for all-cause mortality**	**Normal weight (BMI <25 kg/m^2)**		**Preobese-obese (BMI ≥25 kg/m^2)**	***P* value**
	Total body fat distribution (cut off at **TBF% Tertile 3**)			
	**Normal weight non-obese**	**Normal weight obese**		
Male	<30.5%	≥30.5%		
Female	<37.1%	≥37.1%		
Unadjusted	1.21 (0.90-1.63)	2.34 (1.56-3.52)**	1 (reference)	<0.001
Fully-adjusted	1.07 (0.78-1.47)	1.73 (1.14-2.64)*	1 (reference)	0.029
	Central fat distribution (cut off at **WHtR Quartile 3**)			
	**Normal weight non-obese**	**Normal weight obese**		
Male	<54.3%	≥54.3%		
Female	<54.9%	≥54.9%		
Unadjusted	1.02 (0.88-1.19)	1.89 (1.60-2.23)**	1 (reference)	<0.001
Fully-adjusted	1.17 (1.00-1.37)	1.34 (1.13-1.59)**	1 (reference)	0.003

Values are expressed as hazard ratios and 95% confidence intervals.
Fully adjusted model, adjusted for age, sex, eGFR, Upcr log, diabetes, cardiovascular disease, smoking, cancer, severe liver disease, hypertension, hemoglobin, body mass index, cholesterol log, glycosylated hemoglobin, albumin, CRP ln, and phosphorus.
*$P < 0.05$ compared with reference TBF% group.
**$P < 0.001$ compared with reference TBF% or WHtR group.
Abbreviations: HR, hazard ratio; WHtR, waist-to-height ratio; TBF%, total body fat percentage; NWO: normal weight obesity, BMI: body mass index.

Supplementary Table 4 | Odds ratios for metabolic syndrome stratified by total body fat percentage and body mass index

**OR for metabolic syndrome**		**Total body fat (%)**			
		**Q1**	**Q2**	**Q3**	**Q4**
	Male	<22.2%	22.2-27.4%	27.4-31.9%	>31.9%
	Female	<27.7%	27.7-33.6%	33.6-39.0%	>39.0%
BMI < 25 (Kg/m^2)	Unadjusted	1 (reference)	1.19 (0.80-1.76)	2.07 (1.36-3.15)**	2.76 (1.56-4.88)**
	Fully-adjusted	1 (reference)	1.14 (0.74-1.75)	2.01 (1.25-3.22)*	2.33 (1.24-4.37)*
BMI ≥ 25 (Kg/m^2)	Unadjusted	1 (reference)	1.24 (0.57-2.70)	1.43 (0.69-2.95)	2.01 (0.99-4.07)
	Fully-adjusted	1 (reference)	1.52 (0.64-3.65)	1.57 (0.69-3.59)	2.01 (0.90-4.52)

Values are expressed as hazard ratios and 95% confidence intervals.
Fully adjusted model, adjusted for age, sex, eGFR, Upcr log, diabetes, cardiovascular disease, smoking, cancer, severe liver disease, hypertension, hemoglobin, body mass index, cholesterol log, glycosylated hemoglobin, albumin, CRP ln, and phosphorus.
$^{*}P < 0.05$, $^{**}P < 0.001$ compared with reference TBF% group.
Abbreviations: OR, odds ratio; BMI, body mass index; eGFR, estimated glomerular filtration rate; Upcr, urine protein and creatinine ratio; CRP, C-reactive protein; TBF%, total body fat percentage.
